# Arsenic treatment increase Aurora-A overexpression through E2F1 activation in bladder cells

**DOI:** 10.1186/s12885-017-3253-1

**Published:** 2017-04-18

**Authors:** Yu-Ting Kao, Chin-Han Wu, Shan-Ying Wu, Sheng-Hui Lan, Hsiao-Sheng Liu, Ya-Shih Tseng

**Affiliations:** 1 Department of Microbiology and Immunology, College of Medicine, National Cheng Kung University, Tainan, Taiwan; 2 Institute of Basic Medical Sciences, College of Medicine, National Cheng Kung University, Tainan, Taiwan; 3 Department of Medical Laboratory Science and Biotechnology, College of Medicine and Life Science, Chung Hwa University of Medical technology, Tainan, Taiwan

**Keywords:** Aurora-A, Arsenic, Bladder, Carcinogenesis

## Abstract

**Background:**

Arsenic is a widely distributed metalloid compound that has biphasic effects on cultured cells. In large doses, arsenic can be toxic enough to trigger cell death. In smaller amounts, non-toxic doses may promote cell proliferation and induces carcinogenesis. Aberration of chromosome is frequently detected in epithelial cells and lymphocytes of individuals from arsenic contaminated areas. Overexpression of Aurora-A, a mitotic kinase, results in chromosomal instability and cell transformation. We have reported that low concentration (≦1 μM) of arsenic treatment increases Aurora-A expression in immortalized bladder urothelial E7 cells. However, how arsenic induces carcinogenesis through Aurora-A activation remaining unclear.

**Methods:**

Bromodeoxyuridine (BrdU) staining, MTT assay, and flow cytometry assay were conducted to determine cell proliferation. Messenger RNA and protein expression levels of Aurora-A were detected by reverse transcriptional-PCR and Western blotting, respectively.

Centrosome of cells was observed by immunofluorescent staining. The transcription factor of Aurora-A was investigated by promoter activity, chromosome immunoprecipitation (ChIP), and small interfering RNA (shRNA) assays. Mouse model was utilized to confirm the relationship between arsenic and Aurora-A.

**Results:**

We reveal that low dosage of arsenic treatment increased cell proliferation is associated with accumulated cell population at S phase. We also detected increased Aurora-A expression at mRNA and protein levels in immortalized bladder urothelial E7 cells exposed to low doses of arsenic. Arsenic-treated cells displayed increased multiple centrosome which is resulted from overexpressed Aurora-A. Furthermore, the transcription factor, E2F1, is responsible for Aurora-A overexpression after arsenic treatment. We further disclosed that Aurora-A expression and cell proliferation were increased in bladder and uterus tissues of the BALB/c mice after long-term arsenic (1 mg/L) exposure for 2 months.

**Conclusion:**

We reveal that low dose of arsenic induced cell proliferation is through Aurora-A overexpression, which is transcriptionally regulated by E2F1 both in vitro and in vivo. Our findings disclose a new possibility that arsenic at low concentration activates Aurora-A to induce carcinogenesis.

## Background

Three studies reported an endemic area of blackfoot disease (BFD) in the southwestern region of Taiwan [1–3], and one report showed high levels of arsenic and fluorescent substances in the underground water in this area [4]. Arsenic in the drinking water correlates with high incidence of bladder, lung, renal, and skin cancers in the areas of endemic BFD [5–9]. Furthermore, the correlation between arsenic exposure and occurrence of bladder cancer has been demonstrated by epidemiological studies [8, 9]. This speculation was supported by setting up the tap-water supply system in these arsenic endemic regions and bladder cancer mortality rate reduced afterword [10].

Inorganic arsenic has been defined as a human carcinogen [11]. Natural and anthropogenic sources may release arsenic into the atmosphere. Exposure to arsenic may induce either cell proliferation or apoptosis depending on the dose of arsenic [12]. In addition, the duration of arsenic exposure is another parameter involved in the carcinogenesis.

Mitotic serine/threonine kinase Aurora family consists of Aurora-A, B and C three members with 403 amino acids and the molecular weight of 46 kDa [13]. Aurora-A participates in cell mitosis and meiosis, and proliferation through regulation of cell cycle [14]. In proliferative cells, Both of the mRNA and protein levels of Aurora-A are dynamically expressed at the G1, S, and G2 phase. Briefly, its expression decreased between mitotic exit and entrance of the G1 phase of the next cell cycle [15]. Aurora-A dysfunction may lead to genomic damage including centrosome amplification and chromosomal instability. It further correlates with the tumorigenesis of diverse cancer cells. Vader and Lens reported that Aurora-A can be regulated by transcriptional upregulation or gene amplification [14]. Abnormal Aurora-A dysfunction causes centrosome amplification and transformation of two murine fibroblasts [16, 17], implying that it may play similar role in human cancers. Elevated expression of Aurora-A is associated with diverse clinical pathogenic parameters, including tumor grade, invasion, metastasis, and low overall survival of bladder cancer patients [18].

Comparison of bladder cancer tumor cells from BFD endemic area and other regions reveals higher Aurora-A expression in the former [19]. Arsenic increases the activity of c-*myc* and E2F-1 [20] and selective activation of NF-kB and E2F by low concentration of arsenite in U937 human monocytic leukemia cells [21]. Aurora-A acts as a direct target of E2F3 during G2/M cell cycle progression [22]. Increased E2F1 protein level accompanied with Aurora-A overexpression was detected in breast cancer specimens. Further analysis reveals that Aurora-A increased E2F1 protein stability by suppressing its degradation [23]. Currently, how arsenic-related Aurora-A dysfunctions through gene amplification or epigenic modification remain unknown.

This study aimed to reveal the molecular mechanism of arsenic-induced tumor development. We established an immortalized human uroepithelial cell line model system, and set up a mouse-arsenic exposure model to validate our cell line investigation.

## Methods

### Cell line and culture

The immortalized bladder urothelial E7 cells (ATCC, #CRL-2017) contain HPV E7 oncogene, which binds with phosporylated tumor suppressor RB protein (provided by Nan-Haw Chow; National Cheng Kong University Hospital) [24]. This cell line was maintained in F12 medium (GIBCO, Carlsbad, CA, USA) supplemented with 10% fetal bovine serum at 37 °C in a 5% CO_2_ incubator.

### Arsenic treatment

The immortalized E7 cells were treated with different amount of sodium arsenite (NaAsO_2_; Fluka, St. Louis, MO, USA) for various times and the protein was collected using lysis buffer (50 mM Tris-HCl, pH 7.4, 1% Nonidet P-40, 150 mM NaCl, 0.5% Sodium deoxycholate). RNA was extracted by TRizol™ (Invitrogen, Carlsbad, CA, USA), and genomic DNA was extracted by the commercial kit, YGB100 (RBC Bioscience, Taipei, Taiwan).

### Immunofluorescent assay (IFA)

E7 cells (1 × 10^5^ or 5 × 10^4^/well) were plated in 6-well plates. After incubation with arsenic for one week, cells were fixed with 3.7% formaldehyde for 30 min followed by washing with 1X PBS for 30 min and 0.1% Triton X-100 treatment for 30 min. Cells were washed again with 1X PBS, immersed with blocking buffer (Thermo, Rockford, IL, USA) for 30 min, and then stained with mouse anti-BrdU antibody (#RPN20AB, Amershan Biosciences, Buckigamshire, England), mouse anti-Aurora-A antibody (NCL-L-AK2, Novocastra, Bannockburn, IL, USA) and mouse anti-α-tubulin antibody (Sigma Chemical Co., St, Louis, MO, USA) at 4°Covernight. The next day, cells were washed and stained with Fluirescein (FITC)-conjugated donkey anti-mouse IgG (Jackson ImmunoResearch, West Grove, PA, USA) for 1 h. To stain the nuclear DNA, cells were incubated with Propidium Iodide (PI, 5 μg/ml; Sigma), or Hochest 33,258 (50 ng/ml, Sigma).

### Flow cytometry analysis

Cell cycle distribution was determined by flow cytometry. Cells (1 × 10^5^/well) were plated in 6-well plates. After incubation with different doses of arsenic for one week, cells were collected and fixed with 70% ethanol at −20 °C overnight. The cell cycle distribution was analyzed after PI (40 μg/ml) staining for 1 h.

### Western blotting

Cells were lysed in lysis buffer, and 50 μg of lyset was loaded onto a SDS-PAGE followed by to a PVDF membrane (Millipore, Billerica, MA, USA) transferring. Aurora-A, and β-actin levels were determined by anti-Aurora-A (Cell signaling, Boston, MA, USA) and anti-β-actin (Sigma) antibodies.

### cDNA preparation and RT-PCR

Total RNA (1 μg) was used to prepare cDNA according to the manufacturer’s instructions (Improm-IITM Reverse Transcriptase; Promega, Madison, WI, USA). The cDNA (1 μg) was used for PCR according to the manufacturer’s instructions (YEAtaq DNA polymerase; Yeastern Biotech, Taipei, Taiwan). Primers were used as follows,

Aurora-A(F): GAAATTGGTCGCCCTC;

Aurora-A(R): TGATGAATTTGCTGTGATCC;

18 s rRNA(F): AAACGGCTACCACATCCAAG;

18 s rRNA(R): CCTCCAATGGATCCTCGTTA.

### Promoter activity assay

The plasmids, including pGL2-AAP (provided by Dr. Liang-Yi Hung), pRLTK (at the molar ratio of 10:1) and pCMV-E2F1 (provided by Dr. Ju-Ming Wang), were co-transfected into 1 × 10^5^ E7 cells seeded in a twelve-well plate using Lipofactamin 2000™ (Invitrogen). After arsenic treatment, cell lysate was collected. The lysate in the eppendorf tubes was centrifuged at 13000 rpm at 4 °C for 1 min. LAR II was added to measure the firefly luciferase activity. *Renilla* luciferase activity was determined and used to normalize firefly luciferase activity.

### Chromatin immunoprecipitation (ChIP) assay

ChIP assay was conducted following the manufacture’s protocol (Active Motif). Briefly, after arsenic treatment, cells (5 × 10^7^) were fixed with 1% formaldehyde at RT for 5 min, followed by nuclei extraction. ChIP assay was conducted overnight at 4 °C using 7 μg of DNA and 3 μg of control IgG or E2F1 (C-20, Santa Cruz) after enzyme digestion of chromatin at 37 °C for 30 min. We used protein G magnetic beads (Active Motif) to capture the ChIP product, which was digested with proteinase K after reversion of cross-links for 15 min at 95 °C. The isolated DNA was used as the template for PCR amplification. The primers used for Aurora-A promoter were: site 1 (−268 to −80), sense 5′-TGGTCCGGTTCTCTTGGTAT-3′ and antisense 5′-AAGCTTGACGCATTGGAGAT-3′; site 2 (−104 to +106), sense 5′-CGACGCGTTGGCTCCACCACTTCCGG-3′ and antisense 5′-CCAGGAGCTCAGCCGTTAGAATTCAAAGG-3′ (He, [22]).

### E2F1 silencing using shRNA

The lentivirus of hairpin (sh) RNA against E2F1 (target sequence: 5′-cgtggactcttcggagaactt-3′) was from National RNAi Core Facility of Academia Sinica (Taipei, Taiwan). We infected the bladder cancer E7 cell with this sh RNA for 1 day. We then used 1 μg/ml puromycin for one week to screen stable clones.

### Mice feeding

Four weeks-old female BALB/c mice (Laboratory Animal Center, National Cheng Kung University, College of Medicine, Tainan, Taiwan) were used. All animal experiment protocols were approved by the Laboratory Animal Committee at National Cheng Kung University. Mice were feed with different doses of arsenic (0 and 1 mg/L) in their drinking water. The mice were treated with arsenic containing water for two months. The mice were i.p. injected with BrdU (50 mg/kg) for 24 h before sacrificed. Their bladder, uterus, lung, and kidney were collected for further study.

### Immunohistochemical staining

The paraffin embedded specimens were processed by deparaffinization and rehydration. The slides were incubated in distilled water for 3 min, and immersed in Tris-EDTA retrieval buffer (10 mM Tris Base, 1 mM EDTA, 0.05% Tween 20, pH 9.0). The slides were heated in a microwave for 5 min. Ice bath was used to cool down the slides to RT. The slides were rinsed in 1X PBS for three times 5 min each time, and blocked in 3% H_2_O_2_ (in methanol) for 10 min at RT. The slides were rinsed with 1X PBS for three times, and then immersed in blocking buffer (Thermo) for 30 min. After incubating with the primary antibody [rabbit anti-PCNA (#FL-261, Santa Cruz, Santa Cruz, CA, U.S.A.), mouse anti-BrdU (#RPN20AB) and mouse anti-Aurora-A antibody (NCL-L-AK2)] at 4 °C overnight, the slides were rinsed with PBS for three times followed by incubation with biotin linked secondary antibody (DakoCytomation, LSAB2 System-HRP, Glostrup, Denmark) for 10 min at RT. Again, the slides were rinsed with PBS three times, followed by adding Streptavidin reagent (DakoCytomation, LSAB2 System-HRP, USA) to cover the specimen for 10 min at RT. The slides were rinsed with 1X PBS three times followed by AEC solution treatment for 15 min at RT. The slides were rinsed with distilled water and counterstained with hematoxylin. The slides were then mounted with IHC Mounting Medium (DakoCytomation).

### Statistical analysis

Student’s t test was used to analyze the significance of aurora-A promoter activation in the presence or absence of arsenic.

## Results

### Low concentration of arsenic treatment increased growth rate of immortalized bladder cells

**Fig. 1:**
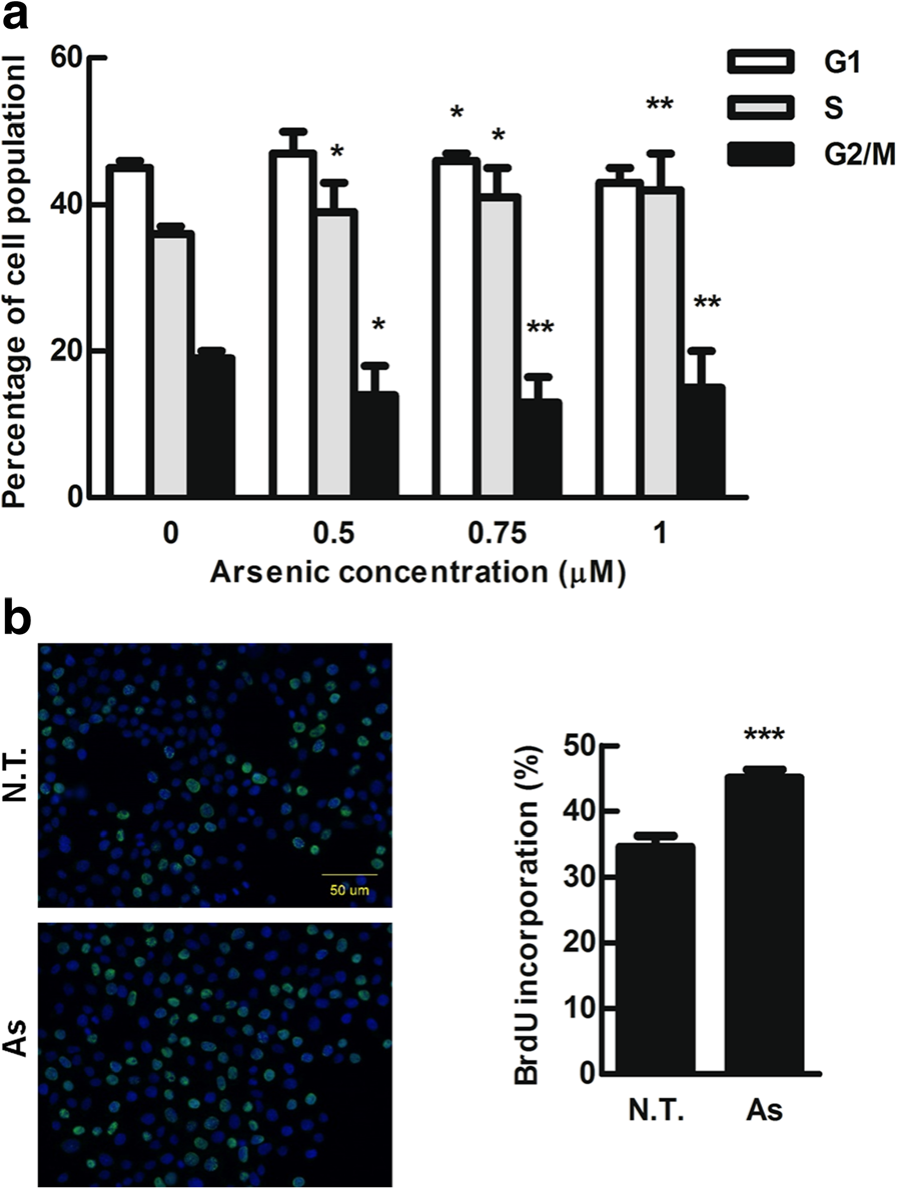
Low-dose of arsenic promotes cell proliferation. **a** E7 cells were treated with indicated concentration of NaAsO2 for one week and the percentage of cell population was detected by flow cytometry. **b** E7 cells were treated with or without arsenic (1 μM) for 1 week. BrdU (10 μM) was added into the cultured medium for 1 h before fixing the cells by 4% paraformaldehyde. BrdU positive cells (*green* color) were stained by BrdU antibody conjugated with FITC (*left* panel). The quantification of BrdU positive-cells was shown in the *right* panel. Data were represented as mean ± S.D. of 2 independent experiments. Student’s t test was used, *p < 0.05, **p < 0.01, ***p < 0.001 compared with non-arsenic treated cells (0 μM)

Long-term exposure of arsenic at low concentration promotes skin cell proliferation and carcinogenesis in vitro and in vivo has been reported [25]. To clarify whether arsenic affects the bladder cancer cells, we dissected the cell population of immortalized bladder urothelial E7 cells at each cell cycle state after low concentration of arsenic (0.5, 0.75 and 1 μM) treatment by PI staining following with flow cytometry analysis. Figure 1a showed that low-dose of arsenic treatments for one week increased cell population at S phase, but decreased cell population at G2/M phase. Furthermore, we used BrdU staining to evaluate effect of arsenic on cell proliferation. After arsenic treatment for one week, the cells with positive BrdU staining were significantly increased, suggesting an increase of cell proliferation (Fig. 1b). Altogether, our results reveal that low dose of arsenic induced cell proliferation by cell population accumulation at S phase of cell cycle.

### Low dose of arsenic treatment induced Aurora-A overexpression followed by multiple centrosome formation

**Fig. 2:**
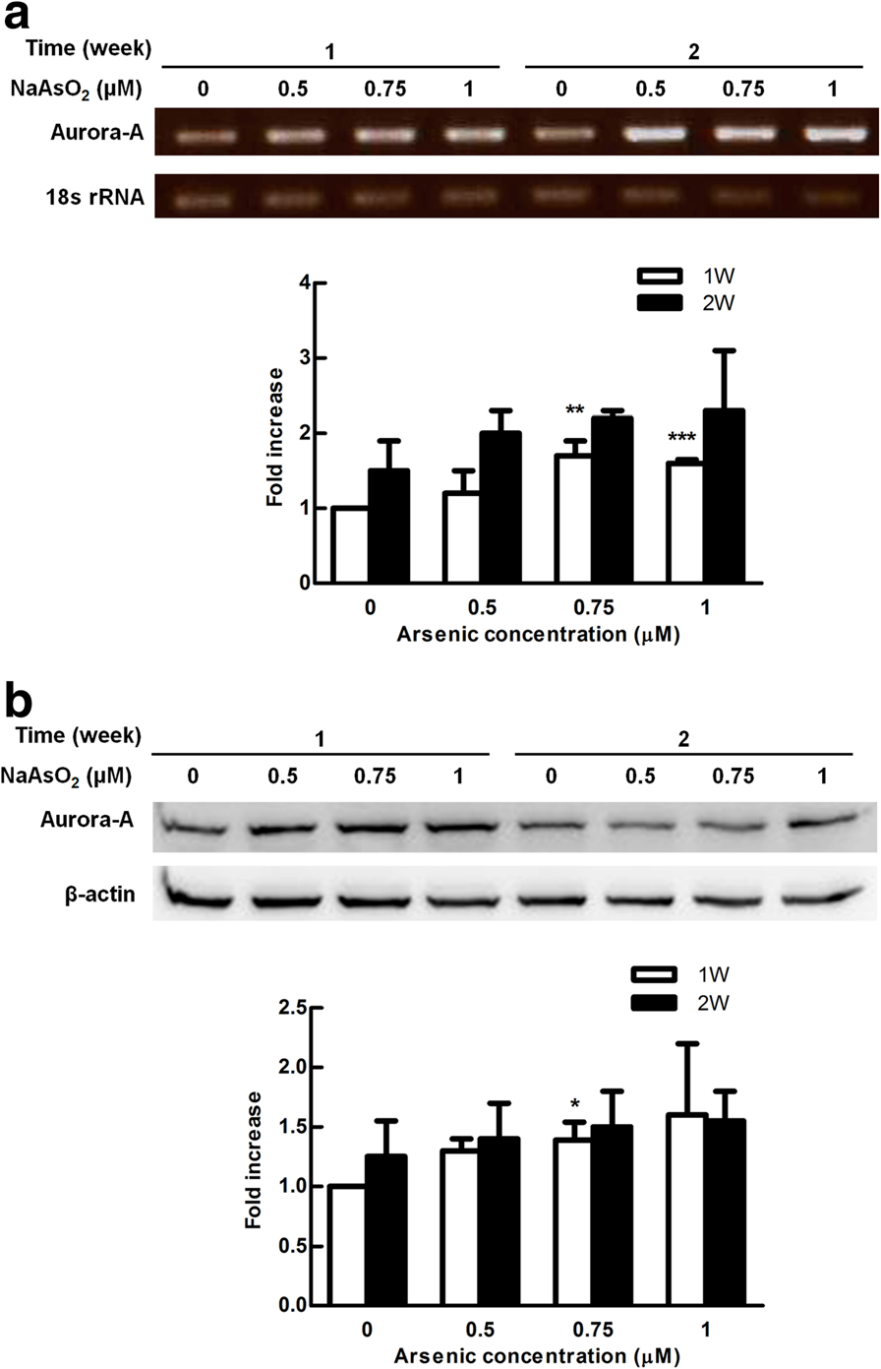
Arsenic promotes Aurora-A expression. E7 cells were treated with indicated concentration of arsenic for one and two weeks, respectively. **a** Total mRNA was purified and analyzed for Aurora-A mRNA expression by RT-PCR. 18 s rRNA was used as the internal control. Quantification of panel A was shown as mean ± S.D. from 3 independent experiments. Student’s t test was used **p < 0.01, ***p < 0.001 (compared to the value of untreated group). **b** Total protein was extracted to evaluate Aurora-A protein expression using anti-Aurora-A antibody by Western blotting. The same blot was re-probed with antibody against β-actin as the loading control; Quantification of panel **b** was shown as mean ± S.D. from 3 independent experiments. Student’s t test was used *p < 0.05 (compared to the value of untreated group)

We previously reported that chronic arsenic exposure leads to increased Aurora-A expression in human bladder cancer [19]. To clarify the effect of arsenic on Aurora-A expression, mRNA and protein levels of Aurora-A were evaluated after arsenic (0.5 to 1 μM) treatment for one and two weeks by RT-PCR and Western blot analysis, respectively. Our results showed that the levels of Aurora-A mRNA (Fig. 2a) and protein (Fig. 2b) were increased as dosage and treatment time increased.

**Fig. 3:**
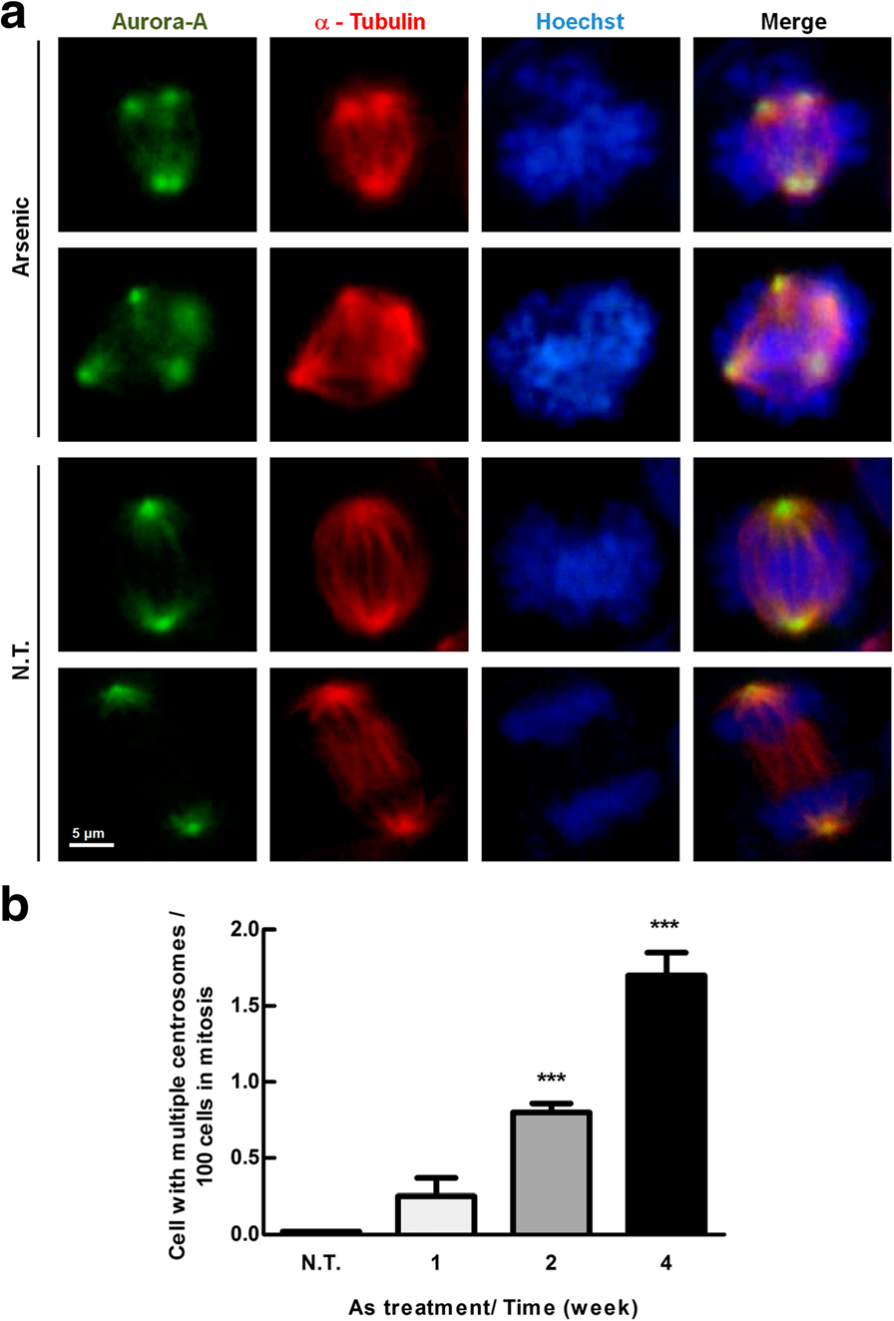
Arsenic exposure caused multiple centrosome formation. **a** E7 cells were treated with arsenic (1 μM) for one week. These cells were fixed and labeled with Aurora-A and α-tubulin antibodies conjugated with FITC (Aurora-A) and PE (α-tubulin), respectively, and finally imaged under confocal microscopy. The Hoechst was used as nuclear counterstain. **b** E7 cells were treated with or without arsenic (1 μM) for one, two, and four weeks, then fixed and labeled as the same as panel (**a**). Quantification of the numbers of multiple centrosomes was normalized by mitotic cells (Aurora-A positive cells). Results are represented in two independent experiments. Student’s t test was used, *p < 0.05, **p < 0.01

Previous studies reveal that high level of Aurora-A induces multiple centrosome formation followed by aberrant cell mitosis process of skin cancer cells, which may trigger tumor formation [25]. To clarify whether arsenic-induced multiple centrosome formation of bladder cancer cells is associated with increase of Aurora-A expression, immortalized bladder urothelial E7 cells were treated with low concentration of arsenic (1 μM) for one to four weeks and then the distribution of Aurora-A and α-tubulin in the cell were investigated by immunofluorescent staining. During mitosis, Aurora-A normally locates at centrosome and spindle poles (Fig. 3a, NT). Differently, abnormal Aurora-A distribution was detected in arsenic-treated E7 cells (Fig. 3a, arsenic). The percentage of cells with more than two centrosomes also increased in a time-dependent manner (Fig. 3b). In summary, our results suggest that arsenic-induced multiple centrosome formation correlates with Aurora-A overexpression in E7 cells.

### E2F1 transcriptionally regulates Aurora-A expression after arsenic treatment

**Fig. 4:**
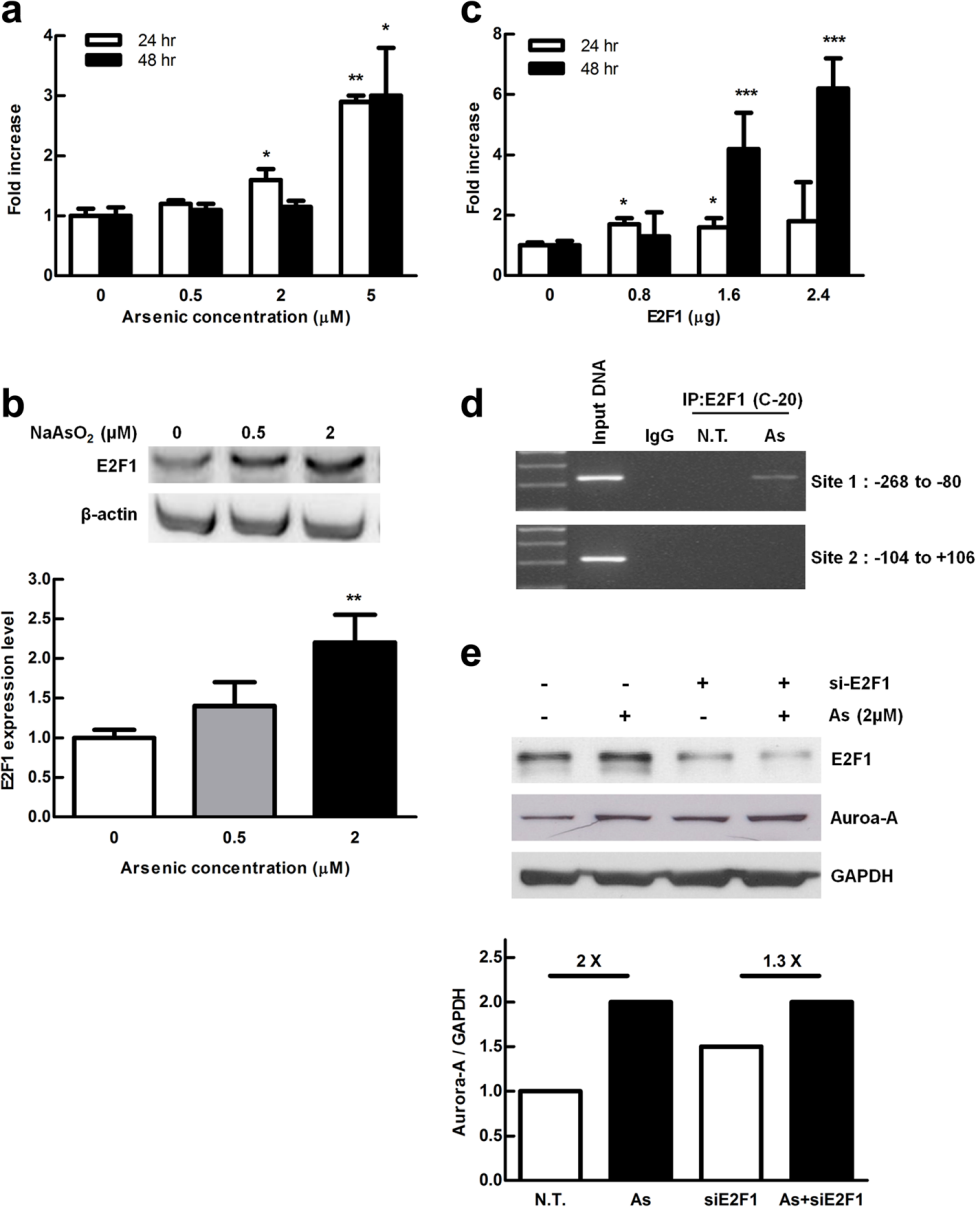
E2F1 was responsible of arsenic exposure induced Aurora-A overexpression in E7 cells. **a** E7 cells were co-transfected with pGL2-AAP plasmid (Aurora-A luciferase promoter reporter) and pRLTK (*Renilla* as an internal control), and treated with arsenic (0, 0.5, 2 and 5 μM) for 24 and 48 h. Data are shown in triplicate as mean ± S.D. of 9 samples from 3 independent experiments. Student’s t test was used *p < 0.05, **p < 0.01 (compared to the value of untreated group). **b** E7 cells were treated with arsenic (0, 0.5 and 2 μM) for 24 h and total protein was extracted to evaluate E2F1 protein expression with anti-E2F1 antibodies by Western blotting. The same blot was re-probed with antibody against β-actin as the internal control. **c** Cells were co-transfected with pGL2-AAP plasmid (Aurora-A luciferase promoter reporter), pRLTK (*Renilla* as an internal control) and different amount of pCMV-E2F1 plasmid which containing full length E2F1 cDNA for 24 and 48 h. The luciferase activity of the cells was evaluated by Dual-Luciferase analysis system. Student’s t test was used as compared to the value of E2F1 untreated one. *p < 0.05, **p < 0.01, ***, *p* < 0.001. **d** The enzymatic-sheared chromatin was pulled down with an anti-E2F1 antibody. The two putative E2F1 binding sites (from −268 to −80 and −104 to +106) within Aurora-A promoter was carried out by PCR. **e** E7 cells were infected with or without lentivirus expressing E2F1 shRNA. After puromycin selection, E2F1 silencing stable cells were treated with 2 μM of arsenic for 2 to 4 days and the protein levels of E2F1 and Aurora-A were evaluated. Quantitative presentation of relative Aurora-A induction by arsenic with E2F1 silencing (sh E2F1) or without E2F1 silencing (wild type E2F1). *P* < 0.05 (compared to the cells without arsenic treatment). The data were analyzed by Student’s t test

Aurora-A genomic aberration including gene amplification and promoter methylation was not detected in arsenic-treated E7 cells (data not shown). Therefore, we further clarified whether arsenic induced Aurora-A expression is through transcriptional regulation. The promoter activity of *Aurora-A* gene was evaluated by a reporter plasmid containing an Aurora-A promoter fused to a luciferase gene, named pGL2-AAP. Our data showed that the Aurora-A promoter activity was elevated after arsenic treatment for 24 h and 48 h when the dose was increased (Fig. 4a). It implies that arsenic transcriptionally regulates Aurora-A expression.

Previous studies reported that E2F transcription factor is activated after arsenic treatment [21]. Here, we found that the protein level of E2F1, a member of E2F family, was increased dose dependently after arsenic treatment for 24 h (Fig. 4b). In addition, Aurora-A promoter activity was induced by E2F1 in a dose-dependent manner at 48 h after co-transfection of the E2F1 (pCMV-E2F1) and Aurora-A reporter (pGL2-AAP) plasmid (Fig. 4c). To confirm arsenic-induced E2F1 directly binds the promoter region of Aurora-A, we carried out the chromatin immunoprecipitation (ChIP) assay using the primer pairs that cover the E2F binding sites proximal to the initiation site of Aurora-A promoter which is critical for Aurora-A activity (He, [22]). After treating with low doses of arsenic for 7 days, a PCR product was amplified by the paired primers covering −268/−80 region of Aurora-A promoter in the immunoprecipitate captured by E2F1 antibody, implying that arsenic-related E2F1 transcriptionally regulates Aurora-A promoter (Fig. 4d).

To further confirm that overexpressed Aurora-A expression is regulated by E2F1, the RNA interference-mediated silence of E2F1 expression was performed. The E2F1 expression levels were greatly reduced as compared with control cells either with or without arsenic treatment (Fig. 4e, upper line, lanes 3 and 4 vs. lanes 1 and 2). However, in the presence of arsenic, Aurora-A expression in E2F1 silencing cells is similar to that in the cells without E2F1 silencing (Fig. 4e, lane 2 vs. lane 4). Silencing of E2F1 seems to be the only blockage of arsenic-related Aurora-A overexpression demonstrated by reduced fold change (Fig. 4e, middle line and quantification diagram: 2× vs. 1.3 ×). Altogether, our results reveal that low concentration of arsenic increased E2F1 expression, which further binds to Aurora-A promoter and increases its expression.

### Aurora-A expression was increased in bladder and uterus of the mice after long-term ingesting arsenic water

**Fig. 5:**
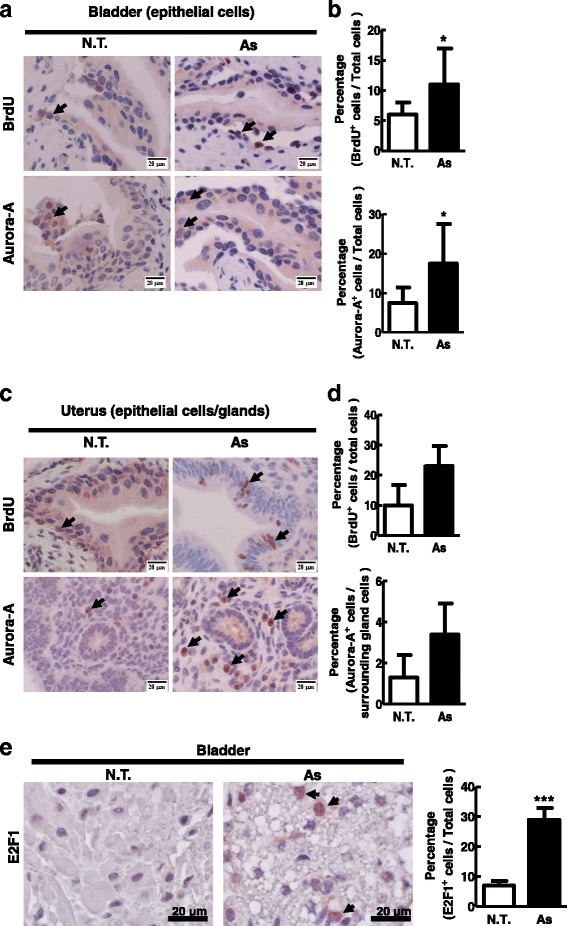
Arsenic increased Aurora-A expression in the bladder and uterus tissues of mice. Four weeks-old BALB/c mice were untreated or treated with arsenic for two months. These mice were i.p. injected with BrdU (50 mg/kg) 24 hr before sacrificing. The bladder (**a**) and uterus (**c**) tissue sections of the mice were treated with BrdU and Aurora-A antibodies respectively, followed by IHC staining. The positively stained cells were quantified in (**b**, bladder) and (**d**, uterus). **e** The sections of the bladders of the mice were treated with E2F1 antibody followed by IHC staining. The scale bar was 20 μm. The data were analyzed by Student’s t test. *: *p*<0.05; ***: *p*<0.001

We established a mouse model to clarify the effect of long exposure of low dose of arsenic on Aurora-A expression and tumorigenesis. Mice were sacrificed and the various tissues were analyzed after treatment with low dose of arsenic (1 mg/litter) in the water for 1 or 2 months. BrdU and Aurora-A protein expression in different tissues were determined by immunhistochemical staining. Both BrdU incorporation and Aurora-A level increased in bladder (Fig. 5a and b) and uterus tissue (Fig. 5c and d) of mice after arsenic treatment for 2 months. Furthermore, E2F1 expression was increased in bladder tissue (Fig 5e). These results suggest that long time low dose of arsenic exposure alleviates Aurora-A expression and cell proliferation on bladder and uterus epithelial in mice. However, no tumor formation was detected in the time period investigated.

## Discussion

The International Agency for Research on Cancer recognizes arsenic and arsenic compounds as group 1 carcinogens, and low concentration of arsenic could promote tumorigenesis [19]. Increased expression of Aurora-A has been strongly associated with the aggressiveness of the tumor, but the factor(s) which induces the overexpression of Aurora-A is still unknown [19]. Based on these evidence, we speculate that Aurora-A might be involved in the regulation of arsenic-induced tumorigenesis. To confirm our hypothesis, we set up the cell line and animal models to clarify the mechanism of arsenic induced Aurora-A overexpression. Our data showed that low concentration of arsenic induces Aurora-A promoter activity, mRNA and protein expression, indicating that arsenic induces Aurora-A overexpression at the transcription level. Aurora-A gene amplification was not detected under long-term, low dose of arsenic treatment. Furthermore, our mouse model reveals that long time exposure of low dose of arsenic could induce Aurora-A expression and uterus epithelial cell proliferation. However, no tumor formation was detected at the time period investigated.

Arsenic increases proliferation of the epithelial cells of various tissues including bladder and skin [26–28]. Cell proliferation depends on an ordered and tightly regulated process known as cell cycle, involving multiple checkpoints which assess extracellular growth signals, cell size, and DNA integrity [28]. In our data, increased S phase population, multiple centrosomes and increased cell proliferation suggest that normal cell cycle progression was interrupted by arsenic at low concentration. Consistent with other’s report. Arsenic delays cell cycle reentry into the G1 phase [29]. Aberrant cell cycle progression is widely detected in many cancer cells. Therefore, the factors responsible for G1/S transition and S phase arrest are possibly involved in arsenic induced tumorigenesis [28]. Among them, E2F family proteins and cyclins (cell cycle related proteins) are the potential candidates.

It is known that phosphorylated RB (p-RB) tumor suppressor can bind with the promoter of E2F family proteins and suppress their activity in normal cells [30]. Here, the immortalized E7 cells containing HPV E7 oncogene, which can bind with p-RB [24], may lead to the activation of E2F1 and increased Aurora-A expression. Nevertheless, the effect of E7 and p-RB interaction on low concentration arsenic upregulation of mRNA and protein expression of Aurora-A in the immortalized uroepithelial E7 cells can be excluded because similar events were detected in another immortalized skin cell line, HaCaT [25].

Similarly, arsenic treatment of the mice for 2 months induced Aurora-A expression in uterus and bladder. Arsenic induces gene amplification and shows carcinogenic effects in humans have been reported [31]. However, Aurora-A gene amplification was not detected in both bladder and skin cells (unpublished data). The possibility that longer time is needed to induce Aurora-A gene amplification cannot be excluded. It is known that inorganic arsenic disturbs the epigenetic regulation of various gene expression [32]. In our study, low-dose arsenic treatment for 4 weeks showed no effect on DNA methylation status of Aurora-A promoter region. A total of ten samples were examined, including immortalized bladder and skin cell lines, arsenic-treated immortalized bladder cells, and clinical skin cancer specimens. The results from Methylation-specific PCR (MSP) and sequencing analysis suggest that all the cytosines in 28 CpG dinucleotides within the promoter examined are free of methylation, indicating that the expression of Aurora-A was not affected by the methylation status of the promoter. The distal region of the promoter needs to be analyzed for possibly arsenic-induced Aurora-A expression, because the analyzed region of Aurora-A is only about 1.5 kb upstream of the promoter,. The other possibility is acetylation. Our preliminary data showed that the acetylation status of lysine-9 and lysine-14 on histone H3 increased after arsenic treatment (data not shown). Whether histone acetylation is involved in arsenic induced tumorigenesis needs further confirmation.

Arsenite exerts dual effects: triggering apoptosis at relatively high concentration, whereas inducing partial differentiation at low concentration in leukemia cells. Protein/DNA array analysis showed that E2F was activated after 6 h exposure to 1 and 10 μM arsenite [21]. Arsenic-increased activity of c-*myc* and E2F-1 has been reported [20]. However, there is no paper discussing the relationship between E2F1 and Aurora-A. Aurora-A acts as a direct target of E2F3 during G2/M cell cycle progression [22]. Not only Aurora-A, but Aurora-B mRNA level is also regulated by cell cycle-dependent element (CDE) and cell cycle-gene homology region (CHR). A subset of E2F family proteins (E2F1 and E2F4) binds to the CDE [33]. In this study and our previous report, we demonstrated that E2F1 increases Aurora-A promoter activity after 24 h arsenic treatment both in bladder and skin cells [25]. In the presence of arsenic, Aurora-A expression in E2F1 silencing cells is similar to that in the cells without E2F1 silencing (Fig. 4e, lane 2 vs. lane 4). It is possible that Aurora-A induction was compromised by E2F3 transcription factor when E2F1 was silenced. It is also possible that silencing E2F1 partly affects arsenic alleviated Aurora-A expression as shown by reduced fold change (Fig. 4e, middle line and quantification diagram: 2× vs. 1.3 ×).

Furthermore, E2F1 could bind Aurora-A promoter to regulate its function. NFκB is a transcription factor which is activated by low concentration of arsenic (1 μM) [21]. Arsenic exposure triggers PI3K/Akt/IKK/NFκB signal cascade which in turn plays essential roles in inducing cyclin D1 expression [34]. Therefore, the relationship between Aurora-A and NFκB deserves further exploration [35].

In our animal model, we investigated bladder and uterus after arsenic treatment because Yoshida et al. reported that chronic arsenic exposure may induce skin, lung, bladder, kidney, liver, and uterus malignancies [36]. Our result showed that after arsenic treatment for 1 month, increase of cell proliferation and Aurora-A expression was detected in glands surrounding cells in uterus but not in bladder and skin (unpublished data). The possible reasons are as follows: first, the time of arsenic exposure is not long enough to induce Aurora-A expression and to increase cell proliferation. Longer exposure time may be necessary for induction of Aurora-A expression in many tissues such as bladder and skin. Second, the time for BrdU uptake is not enough in the bladder tissue, because BrdU was i.p. injected at 4 h before sacrifice. It may need longer time for BrdU uptake [37]. Our speculation was confirmed after extending the arsenic treatment to 2 months and BrdU treatment to 24 h, both the uterus and bladder tissues showed increased cell proliferation and Aurora-A expression. Feeding the mice with arsenic in the drinking water mimics arsenic-induced tumorigenesis in nature. However, arsenic that acts as a co-carcinogen, does not induce tumor formation per se [38]. Therefore, to reveal arsenic induced tumor formation in mice, some initiators must be introduced first or co-incubated with arsenic. Two mice models are proposed. Besides using UV or some known carcinogenic agents [38–40] combined with arsenic, we injected low grade tumor cell line, MR4 (a MEF stable cell line, established in our laboratory), into the mice s.c. and combined with arsenic treatment for 1 month. The tumor formation, cell proliferation, and the protein expressions of Aurora-A between untreated or arsenic treated mice were investigated. Another model is transgenic mice, which carry a specific keratinocyte promoter to drive Ras oncogene expression specifically at epithelial cells.

## Conclusion

This study demonstrated that low concentration of arsenic (≤1 μM) could cause Aurora-A overexpression in bladder cells both in vitro and in vivo. We further reveal that arsenic increases E2F1 expression, which transcriptionally alleviates Aurora-A expression by binding to its promoter.

## Abbreviations

BFD: blackfoot-disease
BrdU: Bromodeoxyuridine
ChIP: Chromosome immunoprecipitation
DMEM: Dulbecco’s modified eagle medium
IFA: Immunofluorescence assay
IHC: Immunohistochemical staining
MTT: 3-(4,5-dimethylthiazol-2-yl)-2–5-diphenyl tetrazolium bromide
RT-PCR: Reverse-transcription PCR
